# Predictors of loss to follow up among HIV-exposed children within the prevention of mother to child transmission cascade, Kericho County, Kenya, 2016

**DOI:** 10.11604/pamj.2018.30.178.15837

**Published:** 2018-06-27

**Authors:** Hudson Taabukk Kigen, Tura Galgalo, Jane Githuku, Jacob Odhiambo, Sara Lowther, Betty Langat, Joyce Wamicwe, Robert Too, Zeinab Gura

**Affiliations:** 1 Field Epidemiology and Laboratory Training Program (FELTP), Ministry of Health, Nairobi, Kenya; 2 Moi University, School of Public Health, Eldoret, Kenya; 3 US Centers for Diseases Prevention and Control (CDC), Division of Global Health Protection (DGHP), Kenya; 4 National AIDS and STI Control Program (NASCOP), Ministry of Health, Kenya; 5 County Government of Kericho, Department of Health, Kericho, Kenya

**Keywords:** HIV, HIV-exposed infant, loss to follow up, Kericho, Kenya

## Abstract

**Introduction:**

HIV-exposed infants (HEI) lost-to-follow-up (LTFU) remains a problem in sub Saharan Africa (SSA). In 2015, SSA accounted >90% of the 150,000 new infant HIV infections, with an estimated 13,000 reported in Kenya. Despite proven and effective HIV interventions, many HEI fail to benefit because of LTFU. LTFU leads to delays or no initiation of interventions, thereby contributing to significant child morbidity and mortality. Kenya did not achieve the <5% mother-to-child HIV transmission target by 2015 because of problems such as LTFU. We sought to investigate factors associated with LTFU of HEI in Kericho County, Kenya.

**Methods:**

A case-control study was conducted in June 2016 employing 1:2 frequency matching by age and hospital of birth. We recruited HEI from HEI birth cohort registers from hospitals for the months of September 2014 through February 2016. Cases were infant-mother pairs that missed their 3-month clinic appointments while controls were those that adhered to their 3-month follow-up visits. Consent was obtained from caregivers and a structured questionnaire was administered. We used chi-square and Fisher's Exact tests to compare groups, calculated odds ratios (OR) and 95% confidence intervals (CI), and performed logistic regression to identify independent risk factors.

**Results:**

We enrolled 44 cases and 88 controls aged ≥3 to 18 months: Cases ranged from 7.3-17.8 months old and controls from 6.8-17.2 months old. LTFU cases' caregivers were more likely than controls' caregivers to fear knowing HEI status (aOR= 12.71 [CI 3.21-50.23]), lack knowledge that HEI are followed for 18 months (aOR= 12.01 [CI 2.92-48.83]), avoid partners knowing their HEI status(OR= 11.32 [CI 2.92-44.04]), and use traditional medicine (aOR= 6.42 [CI 1.81-22.91]).Factors that were protective of LTFU included mothers knowing their pre-pregnancy HIV status (aOR= 0.23 [CI 0.05-0.71]) and having household health insurance (aOR= 0.11 [CI 0.01-0.76]).

**Conclusion:**

Caregivers' intrinsic, interpersonal, community and health system factors remain crucial towards reducing HEI LTFU. Early HIV testing among mothers, disclosure support, health education, and partner involvement is advocated. Encouraging households to enroll in health insurance could be beneficial. Further studies on the magnitude and the reasons for use of home treatments among caregiver are recommended.

## Introduction

Pediatric HIV remains a major preventable disease of global public health concern. In 2015, 2.3 million children aged <15 years were living with HIV and >150,000 new HIV infections in children were reported worldwide [1,2]. Globally, 10-14% of children received HIV treatment, and approximately 30% of HIV-exposed infants (HEI) are reported as lost-to-follow-up (LTFU) from HIV programs yearly [3-5]. The AIDS Progress report indicates that about 50% of the eligible pediatric population were not accessing lifesaving ARVs due to failure to complete treatment because of LTFU [6,7]. Kenya is one of 22 sub-Saharan Africa (SSA) countries that account for over 90% of all pediatric HIV infections, the majority of which result from mother-to-child transmission (MTCT) [1,8]. HEI LTFU is also common in sub-Sahara Africa, Kenya reported LTFU of 16.5-22.1% and 11 other SSA countries reported 27.6-41.5% in the first 3 months after birth in a systematic review of which quantified HEI after 18-months follow up estimated a wide range of 19-85% LTFU [9]. Kenya subscribes to the Joint United Nations Programme on HIV/AIDS (UNAIDS), Global Plan Towards the Elimination of New HIV Infections Among Children by 2015, which seeks to reduce MTCT to below 5% by 2015 [10]. However, Kenya has yet to achieve the target of mother-to-child HIV transmission of ≤5%,having reported over 13,000 new infant HIV infections in 2015 [11]. Despite aggressive plans to ensure early access to efficacious HIV interventions and keep HIV-exposed children in care, children continue to drop out of follow-up care at various points along the HIV prevention of mother to child transmission (PMTCT) cascade [7,9,12]. The PMTCT cascade consists of multiple interventions targeting HIV-positive pregnant mothers before delivery and mother-child pairs after delivery. PMTCT interventions begin with HIV counselling and testing of pregnant mothers at health facilities. During the antenatal care period, nutritional support, diagnosis and treatment of opportunistic infections, and antiretroviral drug prophylaxis are provided to all HIV-positive mothers. Upon delivery, antiretroviral treatment is started for all HIV-exposed newborns, and other services are continued including advice on safer infant feeding, psychosocial support, early HIV infant diagnosis, family support and continued follow up and treatment for mother-infant pairs for 18 months after birth. Complete adherence to these interventions can reduce the MTCT transmission rate from 15-45% to <5% among breastfeeding mothers. However to achieve this reduction, countries must address the problem of LTFU [8,13]. Rural Kericho County (population 832,525, Kenya National Bureau of Statistics ([KNBS 2015]), located in South Rift valley, is one of 47 counties of Kenya, and covers an area of 2479 km2. Kericho County has been implementing PMTCT services since 2002, but despite these efforts, the county did not achieve the mother-to-child transmission national target of < 5% by 2015. In 2014, Kericho County adult HIV prevalence was 4.3%, the percentage of women living with HIV was 4.8%, the mother-to-child HIV transmission rate was 8.9%, and the 6-week infant HIV transmission rate was 6.1% [14-16]. The Kericho County HEI LTFU statistics are not available; however, published studies conducted in Kilifi county, a rural coastal area in Kenya, reported 43% HEI LTFU at 2 months and 65% LTFU at 18-month [17]. Another study in rural Western Kenya reported a HEI LTFU of 7.4% at 3 months and 27.4% at 18-months [18]. Maternal-infant pair socio-demographic factors, facility and community information on reasons for LTFU remains limited. This study aimed to identify factors associated with LTFU among HIV-exposed children in Kericho County, to recommend appropriate policies to guide interventions in the implementation of successful PMTCT programs in Kenya, and to develop hypothesis for further research.

## Methods

**Study design:** We conducted a hospital-based case-control study with case-to-control ratio of 1:2. Cases and controls were matched by hospital and frequency matched by age group using 3-6 months, > 6-12 months and >12-18 months age categories.

**Figure 1:**
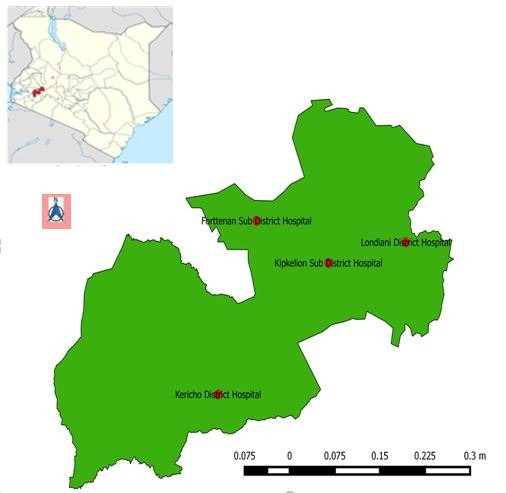
Map of Kericho County showing study facilities, Kenya 2016

**Study population and sampling frame:** Kericho County has 6 sub-counties, with 160 health facilities. Kericho is the larger county hospital, and Kapkatet, Londiani, Sigowet, and Kipkelion are the sub-county hospitals (Figure 1). In 2014, there were 87 county facilities offering pediatric HIV services and PMTCT care and 73 facilities not offering any form of PMTCT services. Study participants were recruited from Kericho, Kipkelion, Fortenan, and Londiani hospitals. The four hospitals register >75% of the HIV-exposed children in the county. HIV-exposed children between 3 and 18 months of age enrolled for HIV care from September 2014 to February 2016 were recruited into the study.

### Definitions

**HIV-exposed infant register:** Ministry of Health HEI register which captures details per birth cohort of HIV-exposed infants seeking services at every facility within the country.

**Case:** All HIV-exposed child 3-18 months old who was enrolled at the hospital for HIV services but had missed appointments for ≥3 months.

**Control:** All HIV-exposed child of same age category as their case, on continued HIV care at the same hospital as case, alive and attending their 3-month appointments as scheduled.

### Sampling

**Sample size assumptions and calculations:** We assumed a two-tailed confidence level at 95%, 80% power, a desired odds ratio (OR) of 0.3 between cases and controls, and a 50% Prevalence [19]. Considering a 15% non-response rate, we estimated a minimum sample size of 132 HEI to be necessary (44 cases and 88 controls) using Fleiss' statistical formula in Epi-Info 7TM.(US Centers for Disease Control and Prevention (CDC)-Atlanta). Potential cases and control subjects were identified from the facility HIV-exposed infant register, categorized by age group, allocated unique identifying numbers, and were selected using a computer generator for random numbers.

**Data collection:** A pre-tested standardized questionnaire was used to collect maternal-infant unique information, socio-demographic data on sex, age, caregiver's level of education, religion, source of income among others, HEI clinical and laboratory information, facility factors including distance to facility, availability of staffs, service delivery health products, facility set-up, and community-related factors to support mother-infant pairs. A trained team of community health extension workers (CHEWs) assisted in recruiting study participants' parents or legal guardians from 6th to 29th June 2016 for in-person interviews.

**Data analysis:** Data analysis was carried out using Epi Info 7 TM(CDC-Atlanta) for the calculation of descriptive statistics. To examine the association between co-variates and LTFU, we calculated chi-square tests, Fisher's exact tests, 95% confidence intervals (CI), and estimated odds ratios (ORs). Adjusted Ors (aOR) were calculated using a logistic regression model with factors having p-values <0.20 during bivariate analysis considered in the model. We used step-wise forward elimination analysis in developing the final model, in which factors with p-values <0.05 were considered significant.

**Ethical considerations:** We obtained written consent from all study participant's parents/legal guardian. Those who were not able to read or write were explained details of the study orally and their ink thumb print was deemed a proof of consent. We obtained ethical approval from Moi Teaching and Referral Hospital Ethics Review Board (IREC 2015/233 No. 1611). This study was determined to be a non-research program evaluation by the U.S. Centers for Disease Control and Prevention Center for Global Health. As an immediate benefit, broader concerted efforts were used for HEIs not receiving antiretroviral drugs and their mothers during and after the study, so that the mothers-infant pairs were traced and encouraged for follow-up, regardless of the status of their enrollment in the study. A generated list of the HEIs who were found to have defaulted and/or LTFU was also shared with facility defaulter tracing teams and program managers for further follow up and tracing.

## Results

**Figure 2:**
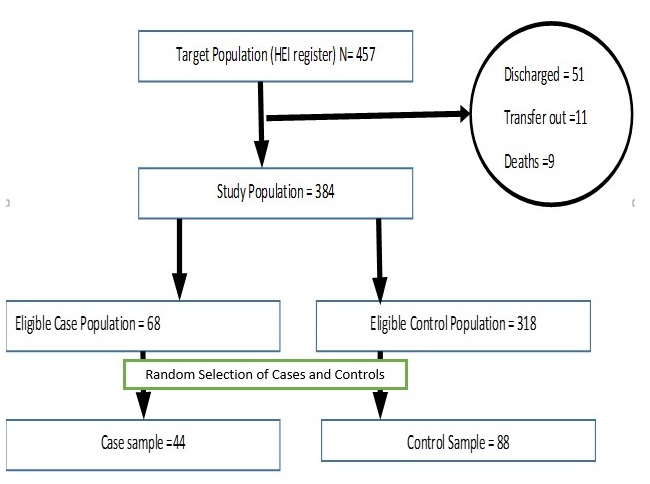
Selection of HIV-exposed infant cases and controls, Kericho County, Kenya, 2016

A total of 457 HEI-caregivers were line-listed; 68 were eligible as cases and 318 as controls. Among the 68 eligible cases, 64 (94.1%) responded; four (5.9%) died. None of the controls declined interviews. A total of 44 HEI cases and 88 paired controls were randomly selected and enrolled in the study (Figure 2). Case ages ranged from 7.30-17.80 months while controls were aged 6.80-17.20 months. Twenty-three (52.3%) cases were male and 48 (54.6%) controls were male. Giving HEIs traditional treatments was among the reasons for failing to adhere to clinic appointments with 31(70.5%) case caregivers report using traditional treatment (Table 1). Of the caregivers interviewed, 124 (94.0%) were mothers of the HEI; 79 (59.9%) were aged 15-30 years (median 29 years, range 19-46 years). A total of 127 mothers (96.2%) had attended at least one antenatal clinic (ANC) visit (Table 2). A majority, 25 (56.8%) of the case caregivers reported not wanting their partner/spouse to know the HIV status of the HEI. (Table 3). A total of 94 (71.2%) HEI mothers were reported to have delivered in health facilities (Table 2). HEI delivered at home were significantly more likely to be LTFU than those delivered in a facility (OR=2.35[CI 1.07-5.35]).Compared by infant weight, there was no significant difference between the cases and controls. There was no difference between cases and controls by facility delivery type (Table 1). Cases were significantly more likely to have had a hospitalization after birth than controls (OR=5.41[CI 1.90-14.61]). The reported use of traditional treatments among HEI was associated with LTFU (OR=5.61[CI 2.71-12.62]).As per the national PMTCT policy and guidelines recommendation, HEIs are to receive ART prophylaxis at birth till they are 12 weeks old. We found no significant difference between LTFU cases versus those HEI who maintained their appointments relating to receipt of infant ARV prophylaxis for the first 12 weeks since birth (Table 1). Of the HEI aged > 6 months, 30 (22.7%) had a mid-upper arm circumference (MUAC) below 12.0 cm. LTFU children were more likely to be malnourished HEIs than those not LTFU (OR=4.30[CI 1.86-10.31]) (Table 1). In multivariable analysis, LTFU cases' caregivers were more likely than controls' caregivers to have fear of knowing HEI status (aOR= 12.71[CI 3.21-50.23]), lack knowledge that HEI are followed for 18 months (aOR= 12.01[CI 2.92-48.83]), avoid partners knowing their HEI status (aOR= 11.32[CI 2.92-44.04]), and use traditional medicine (aOR= 6.42[CI 1.81-22.91]).Factors reducing the odds of LTFU included knowledge of mothers' pre-pregnancy HIV status (aOR= 0.23[CI 0.05-0.71]) and having household health insurance (aOR= 0.11[CI 0.01-0.76) (Table 3, Table 4).

**Table 1:** Socio-demographic Characteristics, Clinical Information of HIV-exposed Infants (HEI), Kericho County, Kenya, 2016

Variable	Cases n (%)	Controls n (%)	p-value
**Sex of HIV-exposed infant(HEI)**			
Male	23(52.3)	48(54.6)	0.197
Female	21(47.7)	40(45.4)	
**Age category(HEI)**			
3-12 months	18(40.9)	33(37.5)	0.174
≥ 12-18 months	26(59.1)	55(62.5)	
**Place of HEI Delivery**			
Home (unskilled delivery)	18(40.9)	20(22.7)	0.030
At Health Facility	26(59.1)	68(77.3)	
**Health facility where HEI is born**			
Government owned	24(92.2)	56(82.3)	0.339
Faith based/ Private owned	02(07.8)	12(17.7)	
**HEI weight (within 7 days since delivery)^#^**			
< 2500g	09(20.5)	11(12.5)	0.231
≥ 2500g	35(79.5)	77(87.5)	
HEI Hospitalized at least once since birth	14(31.8)	07(07.9)	<0.001
HEI Ever used home/traditional treatments	31(70.5)	26(29.6)	<0.001
**Infant ART prophylaxis from birth to 12 weeks**			
Adhered to prophylaxis as per schedule	37(84.1)	78(88.6)	0.464
Defaulted or never started prophylaxis	07(15.9)	10(11.4)	
LMUAC measurement for HEI > 6 months	18(43.9)	13(16.9)	0.001
**Immunization status as per KEPI schedule**			
Missed at least one appointment	08(18.2)	06(06.8)	0.081
Immunization as per schedule	36(81.8)	82(93.2)	

HEI – HIV-exposed infant LMUAC- Left Mid Upper Arm Circumference g- Gram

KEPI- Kenya Expanded Program for Immunization % - Percent

ART- Anti-retroviral therapy

(Within a week)^#^- Weights recorded within 7 days were considered in analysis, those born at home reported at the facilities within the week

**Table 2:** Socio-demographic characteristics, clinical information of the HIV-exposed Infants (HEI) Care-givers, Kericho County, Kenya, 2016

Variable	Cases n (%)	Controls n (%)	p-value
**Interviewed HEI parents**			
Male (father)	01 (02.3)	07 (07.9)	0.198
Female (mother)	43 (97.7)	81 (92.1)	
**Age of caregiver**			
< 30 years	32 (72.7)	47 (53.4)	<0.001
≥ 30 years	12 (27.3)	41 (46.6)	
**ANC attendance by HEI mothers**			
Attended at least one ANC visit	43 (97.8)	84 ( 95.5)	0.410
Never attended ANC visit	01 (02.2)	04 (04.5)	
**Caregiver Educational level**			
< Primary (< 8 years of education)	14 (31.8)	22 (25.0)	0.204
Primary complete (> 8 years of education)	30 (68.2)	66 (75.0)	
**Caregiver’s Religion**			
Christian	42 (95.5)	85 (96.6)	0.108
Muslim	01 (02.3)	03 (03.4)	
**Mother’s employment**			
Formal employment	05 (11.4)	25 (28.4)	0.028
Informal employment	39 (88.6)	63 (71.6)	
**Partner/spouse source of income**			
Formal employment	06 (22.2)	11 (15.7)	0.452
Informal employment	21 (77.8)	63 (71.6)	
**Marital Status**			
Single/Separated	21 (47.8)	18 (20.5)	0.003
Married	23 (52.2)	70 (79.5)	
**Household(Family) size**			
Less than 5 members	58 (65.9)	26 (59.1)	0.444
Equal/more than 5 members	30 (34.1)	18 (40.9)	
Households with ≥ 2 children aged <5 years	19 (43.2)	22 (25.0)	<0.001
Having a family health cover (NHIF)*	05 (11.4)	25 (28.4)	<0.001

HEI- HIV-exposed infant ANC- Antenatal Care NHIF*- National Health Insurance Fund

**Table 3:** HIV-exposed infant caregiver factors associated with loss to follow up, Kericho County, Kenya, 2016

Associated Factor	Cases n (%)	Controls n (%)	OR	95% CI	aOR	95% CI
Age < 30 years	32 (72.7)	47 (53.4)	2.31	1.11-5.12		
In Single or separated marital relationship	21 (47.8)	18 (20.5)	3.42	1.62-7.81		
Household with a Health insurance cover(NHIF)*	05 (11.4)	25 (28.4)	0.23	0.13-0.72	0.11	0.01-0.76
Lacking knowledge on HEI follow up(at least 18 months)	31 (70.4)	20 (22.7)	8.12	3.51-18.44	12.01	2.92-48.83
Attending less than four antenatal visits	32 (72.7)	30 (34.0)	5.23	2.31-11.91		
Not wanting their partner know HEI HIV exposure status	25 (56.8)	13 (14.7)	7.51	3.31-17.61	11.32	2.92-44.04
History maternal ARV prophylaxis	23 (52.3)	87 (98.8)	0.22	0.01-0.12		
Fear knowing HEI HIV outcome status	27 (61.3)	17 (19.3)	6.61	3.01-14.82	12.71	3.21-50.23
Reporting taking alcohol	09 (20.4)	07 (07.9)	2.91	1.12-8.61		
Knowing their HIV status before getting pregnant	14 (31.8)	64 (72.7)	0.12	0.07-0.33	0.23	0.05-0.71
Reporting receiving health education on maternal HIV transmission	27 (61.4)	80 (90.9)	0.21	0.06-0.45		
Sending SMS reminders to male vs to female caregivers	13 (43.3)	34 (75.6)	4.13	1.51-10.91		
Reporting exclusive breastfeeding as culturally accepted	11 (25.0)	75 (85.2)	0.32	0.07-0.41		
Disclosure of HIV status to partner/spouse	13 (29.5)	61 (69.3)	0.23	0.10-0.41		

HEI- HIV-exposed Infant n- Number of cases or controls % - Percent OR – Odds Ratio CI - Confidence Interval aOR- Adjusted Odds Ratio NHIF*- National Health Insurance Fund **ARV- Antiretroviral drug**

**Table 4:** HIV-exposed Infant Factors Associated with Loss to Follow up, Kericho County, Kenya, 2016

Associated Factor	Cases, n (%)	Controls n (%)	OR	95% CI	aOR	95% CI
Home (unskilled) delivery	18 (40.9)	20 (22.7)	2.35	1.07-5.35		
Ever hospitalized since birth	14 (31.8)	07 (07.9)	5.41	1.90-14.61		
Ever used or using traditional medicines	31 (70.5)	26 (29.6)	5.61	2.71-12.62	6.42	1.81-22.91
Left mid upper arm circumference measurement (children > 6 months)	18 (43.9)	12 (13.6)	4.30	1.86-10.31	6.21	1.43-28.13
Household family size ( ≥ 5 members)	30 (34.1)	18 (40.9)	8.33	3.61-18.92		
Household with children ≥ 2 aged ≤ 5 years	19 (43.2)	22 (25.0)	2.34	1.16-4.93		

n- Number of cases or controls % - Percent OR- Odds Ratio aOR- Adjusted Odds Ratio

CI- Confidence Interval HEI- HIV-exposed Infant

ART- Antiretroviral therapy KEPI- Kenya Expanded Program for Immunization

(Within a week) ^#^- Weights reported within 7 days were considered for analysis, those born at home reported at the facilities within the week

## Discussion

We found that HEI clinic follow-up was significantly undermined by non-disclosure, fear and its related negative attitudes towards HIV among mothers, partners, or spouses. We also found that a lack of adequate understanding by caregivers on the need to continue HEI clinic scheduling, and the use of home or traditional therapies among mother-infant pairs. HIV testing of mothers before pregnancy and enrolling households into family health insurance programmes reduced HEI LTFU. The increased LTFU among children whose caregivers had reservations in disclosing their child's HIV exposure status to partners, and those who feared knowing HIV status of their infant reveals current challenges in sharing personal HIV information with partners and family members [13]. This may be due to the perceived or real effects of fear, stigma, and discrimination among mothers living with HIV. Despite the existence of HIV/AIDS epidemic for over three decades and common knowledge and widespread information on HIV/AIDS, stigma remains an important challenge. Mothers in Kenya who are HIV-positive are reluctant to share their child's or their own HIV results with spouses due to the possibility of violence, fear of their partners' unexpected reactions, household conflicts, fear of losing their partners, or the perceived stigma by family, making them believe they will be ignored, isolated and blamed for the child ailments [20]. Studies in rural Zambia [21] and Malawi had similar findings [22]. The lower proportions of HIV disclosure to the male partners by the HIV-positive female partners' highlights reported low male partner involvement which may explain also the low levels of disclosure and partner testing in various PMTCT programs in most SSA countries. Findings from Kenya, Malawi, and Ethiopia show that a majority of male partners are rarely involved in PMTCT programs, likely due to the non-disclosure by partners [23-25]. To improve disclosure among HIV-positive mothers, integrated approaches are needed among stakeholders that target HIV-infected mothers, their partners, and the general public to raise awareness. Sustained advocacy, communication and social mobilization activities on disclosure, reducing stigma, risk reduction behaviors, empowering partners psychosocially and on reducing fear among the populace on matters HIV and related interventions should be strengthened [26]. HIV stigma reduction and openness among HEI caregivers and the community on matters related to HIV can reduce chances of LTFU. Accordingly, HIV-exposed or infected infants should continuously access cotrimoxazole prophylaxis, needed antiretroviral and other supportive HIV care because without this, most infants die before their second birthdays respectively [27]. Parents' lack of knowledge that HEI should receive follow-up care for 18 months despite initial HIV-negative polymerase chain reaction (PCR) results could influence a caregiver's decisions on continuing appointments for HEI who appear healthy.

Findings from rural northern Uganda and South Africa demonstrated that HIV-infected mothers who knew the duration and number of the visits, the reasons for not missing visits, consequences of missing clinic visits, and the reason why their infants were being followed-up, more frequently attended clinic appointments and had successful follow-up outcomes [19]. A clear understanding motivates caregivers so that they are aware of the short or long-term potential benefits of following treatments. A hospital-based case-control study in Cameroon found that mothers who had no formal education were significantly more likely to be lost to follow-up with HIV care programs compared to those with formal education [28]. Educated mothers have better attitudes and practices concerning their children and own health. They are more likely to understand clinical concepts and the language used at healthcare settings. In our study, level of education among cases and control was not statistically significantly associated with LTFU, which could have been because we recruited most of our study participants from referral health facilities serving a cosmopolitan population. It is possible that language used by health care workers (HCWs) during counselling and educating mother-infants pairs might be too complex for caregivers to understand. The patient-HCW communication forms may limit the messages shared with HEI caregivers. Also, the time taken at consulting clinic may have been short or the caregivers did not appreciate the messages they receive at the health facilities. We could not assess these parameters since such variables were not part of our questionnaire. Another study in Kenya showed that poor perception of the patient-clinician communication by clients attending HIV services is associated with lower clinic attendance [29] Mothers who knew and shared their HIV status with their before pregnancy were less likely to have their infant LTFU. Findings from studies done in Ethiopia and in other low HIV prevalence settings in Central Asia and Eastern Europe [30,31] showed that knowledge of maternal HIV prior to pregnancy allows HIV-infected mothers to be better prepared to receive their infants' test results. It may be that they might have had adequate time to be taken through counseling and living positively with HIV, hence have a higher acceptance going forward. There is a higher chance that mothers who knew their HIV status are more likely to have been better prepared psychologically, share their own HIV and HEI status with partners and family, belong to support groups, take maternal ARV prophylaxis, and be more familiar with and adhere to clinician instructions. In our study, these factors were found to help HEI-mother pairs to continue attending clinic appointments. Mothers with early knowledge of their HIV status tend to have better overall health compared to those who get diagnosed during antenatal, labor and delivery and during post-natal periods. They learn to adapt and accept to live positively, have greater motivation to take care for their HEI.

Other studies in South Africa [32] and Uganda [33] have demonstrated that HIV-infected mothers and their exposed children who have adequate health information have a lower risk of LTFU. A study in Western Kenya [3] and in Malawi [6] showed that malnourished HIV exposed or infected children were less likely to attend scheduled visits. This is consistent with our findings that showed a significant association of under nutrition with loss to follow up among HEI who had a mid-upper arm circumference below the accepted baseline for children aged > 6 months. This could explain some of the health outcomes facing LTFU HEI. The finding on association of under nutrition and LTFU had limitations; we did not collect data on income, breast feeding practices, weaning practices, types of food and food security at the household level. This simple clinical marker, undernutrition, could be useful for health care workers in predicting HEIs who could easily be lost to follow up. It remains unknown what happens to HIV-exposed children LTFU within the household. Mother-HEI pairs might be using traditional home treatments. However, as a limitation, the study could not determine the magnitude on the use of home treatment among HEI. The use of home treatments puts HIV-exposed children at risk of progression of disease, malnutrition, illness and death, even if they are not HIV-infected. LTFU and the associated use of home treatment could be demonstrated by the four deaths that were recorded during this study. The four deaths had not been notified to the health facilities. Households that had a family health insurance cover were more often compliant with mother-infant clinic appointments. This finding remains unclear since HIV services at our study sites were not charged. In addition, since June 2013, the Government of Kenya waived all maternity related fees [34]. We did not expect cost-related factors to be a barrier to not seeking services. Having a health insurance cover could be a proxy indicator for other socio-economic determinants, or health-seeking behavioral differences. A systematic review of over 116 studies in SSA [35] suggests that social factors such as lack of income and being unemployed are associated with LTFU. Such social factors could explain a household's ability to have a health insurance plan.A study in a pediatric health program in Ghana showed that apart from registering households into the country's national health insurance scheme and households benefiting from cash transfer programs,belonging to such programs had a facilitative role among mothers to continue taking their children to the health facilities for regular checkups leading to overall improvement of child health indicators [36,37].

Our study had some limitations; nearly all of the HEI caregivers interviewed were mothers, which prevented us from ascertaining the fathers' role, which might be associated with HEI LTFU. The scheduling of clinical appointment dates for some HIV-exposed infants within the HEI register was not strictly as per guidelines, especially for infants who were sick or were being followed for other co-morbidities. For example, for ill children scheduled for visits at <3 months of age, misclassification of their true outcome status might occur. Since we recruited study participants from high-volume referral facilities,our findings might not be generalizable. However, recruitment from different centers and the cosmopolitan population of the study participants and the consistency of findings with that of other studies from similar settings highlights the valuable insights that could allow a better understanding of the predictors of HEI loss to follow up. In addition, a strength of this evaluation is that after these HEIs follow-up efforts, the Kenya HIV estimates and the Kenya AIDS Response Progress Report found that Kericho County had an improvement in maternal ART and, infant ART utilization and a reduction in the number of HEIs LTFU [38,39]. These achievements in 2016 occurred simultaneously with innovative HIV strategies within the 2014 UNAIDS 90-90-90 strategy, to better control the HIV epidemic, and other Kenyan specific campaigns which focused on HIV infected women and children. Caregivers' intrapersonal, interpersonal, community and health system factors remain crucial towards HEI retention. Promoting early HIV testing among mothers, disclosure support, health education, and partner involvement is advocated. Encouraging households to enroll in health insurance could be beneficial. Further studies on the magnitude and the reasons for use of home treatments among caregivers are recommended.

## Conclusion

Caregivers' intrinsic, interpersonal, community and health system factors remain crucial towards reducing HEI LTFU. Early HIV testing among mothers, disclosure support, health education, and partner involvement is advocated. Encouraging households to enroll in health insurance could be beneficial. Further studies on the magnitude and the reasons for use of home treatments among caregiver are recommended.

### What is known about this topic

- The elimination of HIV infection among HIV-exposed infants is achievable and remains of public health importance globally.
- Loss to follow up of caregivers and their HIV-exposed infants has an impact in elimination of HIV interventions.

### What this study adds

- Caregivers' use of traditional medicine continues to significantly contribute to HIV-exposed infant loss to follow up hence hampering HIV elimination targets in Kenya.
- Hospitalised HIV exposed infants (HEIs) are prone to loss to follow up (LTFU), contributing to prevention of mother to child HIV transmission (PMTCT) missed opportunities.
- Social safety nets like encouraging HEI care givers to enrol in health insurance programs may improve retention among HEI and there is need to carry out a quantitative study to determine the magnitude on the use of traditional treatment among HEI.

## Competing interests

The authors declare no competing interests.
